# Drastic decline in sera neutralization against SARS-CoV-2 Omicron variant in Wuhan COVID-19 convalescents

**DOI:** 10.1080/22221751.2022.2031311

**Published:** 2022-02-16

**Authors:** Chengbao Ma, Xianying Chen, Fanghua Mei, Qing Xiong, Qianyun Liu, Lianghui Dong, Chen Liu, Wenjing Zou, Faxian Zhan, Bing Hu, Yingle Liu, Fang Liu, Li Zhou, Junqiang Xu, Yongzhong Jiang, Ke Xu, Kun Cai, Yu Chen, Huan Yan, Ke Lan

**Affiliations:** a State Key Laboratory of Virology, Institute for Vaccine Research and Modern Virology Research Center, College of Life Sciences, Wuhan University, Wuhan, People’s Republic of China; b Hubei Center for Disease Control and Prevention, Wuhan, People’s Republic of China

**Keywords:** SARS-CoV-2, Omicron variant, Delta variant, immune escape, fusion

## Abstract

Global concern has been raised by the emergence and rapid transmission of the heavily mutated SARS-CoV-2 Omicron variant (B.1.1.529). So far, the infection features and immune escape ability of the Omicron variant have not been extensively studied. Here, we produced the Omicron pseudovirus and compared its entry, membrane fusion, and immune escape efficiency with the original strain and the dominating Delta variant. We found the Omicron variant showed slightly higher infectivity than the Delta variant and a similar ability to compete with the Delta variant in using Angiotensin-converting enzyme 2 (ACE2) in a BHK21-ACE2 cell line. However, the Omicron showed a significantly reduced fusogenicity than the original strain and the Delta variant in both BHK21-ACE2 and Vero-E6 cells. The neutralization assay testing the Wuhan convalescents’ sera one-year post-infection showed a more dramatic reduction (10.15 fold) of neutralization against the Omicron variant than the Delta variant (1.79 fold) compared with the original strain with D614G. Notably, immune-boosting through three vaccine shots significantly improved the convalescents’ immunity against the Omicron variants. Our results reveal a reduced fusogenicity and a striking immune escape ability of the Omicron variant, highlighting the importance of booster shots against the challenge of the SARS-CoV-2 antigenic drift.

## Introduction

The SARS-CoV-2 is continuously evolving and generating various variants with distinct features. The spike mutations are closely monitored, given their impact on viral transmission and immune escape. Different spike mutations emerged and became dominant in the emerged variants of concern (VOC), such as the representative mutations D614G, N501Y, E484Q/K, L452R, P681R in the Alpha, Beta, Gamma, and Delta variants [1]. The emergence of the Omicron variant (B.1.1.529) was firstly reported on November 24, and it had been designated as the fifth variant of concern (VOC) by the World Health Organization (WHO) on November 26 [2,3]. The Omicron variant has been reported to be more transmissible but less pathogenic than the current dominant Delta variant [4]. More time and more clinical studies are necessary to assess the risk of the Omicron infection. Indeed, fatal Omicron variant infection cases have been reported recently in multiple countries.

The “weird” Omicron variant is characterized by a large number of mutations in the spikes, including 30 point mutations, three deletions, and one insertion. The 15 receptor-binding domain (RBD) mutations include the previously characterized immune escape-related mutations such as site K417, T478, and E484 and N501, but many are less studied. The heavy mutation may alter the receptor interaction modes, eliminate the activity of many monoclonal antibodies, and compromise the protection of immunity triggered by vaccines or prior infections [5]. The infection features of the Omicron variant have not been extensively studied. However, increasing evidence shows the Omicron variant has extraordinary immune evasive ability to weaken the effectiveness of therapeutic monoclonal antibodies, and sera from the convalescents or the vaccine recipients [6–13]. Until now, the impact of Omicron mutations on the sera neutralizing activity has not been extensively studied in convalescents with a larger cohort.

As the first known epicentre of COVID-19, Wuhan has a group of earliest convalescences infected by the original strain (wild type strain) [14]. In a previous study, we collected 248 sera samples from Wuhan convalescents one-year post-infection, who were absent from Delta and Omicron variants contact and not vaccinated before sample collection [15]. We showed that the humoral immunity of prior infection was well-maintained up to one year, but the protection was partially compromised by the mutations of the emerged variants [16]. It is of public interest that how potent the established anti-SARS-CoV-2 immunity in these earliest convalescences can protect them from the threat of the Omicron variant, and how much can they benefit from the vaccination and booster shots. Here, we further evaluated the neutralizing activity of these sera samples against the Omicron variant.

## Materials and methods

### Cell culture

Vero-E6 cells (ATCC, CRL-1586), BHK-21(ATCC, CCL-10), and HEK293T (ATCC, CRL-1168) were cultured in high-glucose Dulbecco's medium (DMEM; Monad) supplemented with 10% fetal bovine serum (FBS) and 1% penicillin/streptomycin. BHK-21-hACE2 (clone 7) stably expressing human ACE2 was constructed by lentiviral transduction in the lab, maintained with DMEM+10% FBS with puromycin at 1 μg/ml. I1-Hybridoma (CRL-2700) producing a VSV-neutralizing antibody recognizing the VSV glycoprotein was cultured in Minimum Essential Medium with Earle's salts, and 2.0 mM L-Glutamine (MEM; Gibco) replenished with 10% FBS. All the cells were passaged every 2–3 days using Trypsin-EDTA (0.25%, GIBCO).

### Sample collection

One of our previous studies described the details of the 180 sera of Wuhan COVID-19 convalescents collected around one-year post-infection [16]. Briefly, 248 eligible individuals with symptom onset between 2020-1-1 and 2020-3-26 were recruited. Their blood samples were collected from 2021-1-19 to 2021-1-26 without any vaccination. The three sera from the convalescents after three shots of inactivated vaccine (Vero) were collected on 2021-12-23, around 10–43 days after the third vaccination. The collections were carried out through HUBEI PROVINCIAL CENTR FOR DISEASE CONTROL AND PREVENTION and HUBEI PROVINCIAL ACADEMY OF PREVENTIVE MEDICINE (HBCDC) with written consent under appropriate Institutional Review Boards approval (2021-012-01) and were deidentified. Sera were heat-inactivated at 56°C for 30 mins before neutralization assay.

### Plasmids

pCAGGS-SARS-CoV-2-S-dc-D614G expressing WT spike protein with D614G was generated by overlapping-PCR based mutagenesis using pCAGGS-SARS-CoV-2-S-C9 (gifted from Dr. Wenhui Li, National Institute of Biological Science, Beijing, China. Wuhan-Hu-1, GenBank MN908947.) as a template and cloned into pCAGGS vector with C-terminal 18 amino acid (aa) truncation to improve VSV pseudotyping efficiency. The DNA sequences of human codon-optimized S proteins from SARS-COV-2 S of the Delta (GISAID: EPI_ISL_2378732) and Omicron (GenBank: OL672836.1) variants were commercially synthesized and cloned into pCAGGS vector with the C-terminal 18 aa truncation.

### SARS-CoV-2 pseudovirus production and quantification

The VSV-ΔG-based SARS-CoV-2 pseudoviruses were packaged with spike proteins from the D614G and SARS-CoV-2 variants according to a published protocol with slight modification [17]. 293T cells in a 10 cm dish were transfected with plasmids expressing different SARS-CoV-2 variants S proteins through the Gene Twin (Biomed, China) according to the manufacture’s instructions. After 4–6 h (hrs), the medium was replaced by fresh DMEM with 10% FBS. 24 h later, the transfected cells were incubated with 2 ml VSV-dG-fLuc, VSV-dG-GFP or VSV-dG-mcherry (1 × 10^6^ TCID50/ml, rescued and amplified in the lab) diluted in DMEM for 4 h at 37°C, and then replenished with fresh medium (DMEM with 10%FBS) containing monoclonal antibody targeting VSV glycoprotein (I1-hybridoma, cultured supernatant, 1:20 dilution). 24 h later, the SARS-CoV-2 pseudovirus-containing supernatant was collected and centrifuged at 4000 rpm for 10 mins, prepared in aliquot, and stored at −80°C. The 50% tissue culture infectious dose (TCID50) of different SARS-CoV-2 pseudovirus was determined by a serial-dilution based infection assay in a BHK-21 over-expressing hACE2 cells and calculated according to the Reed-Muench method.

### SARS-CoV-2 pseudovirus infection and neutralization assay

The sera were diluted, transferred into 96-well white flat-bottom culture plates, and then mixed with the SARS-CoV-2 pseudoviruses (1 × 10^5^ TCID50/well). After 30 mins incubation at 37°C, the trypsinized BHK-21-hACE2 cells were added into the 96-well white flat-bottom culture plates with viruses/serum samples at a density of 2 × 10^4^/well. After 16 h, the medium of the infected cells was removed and lysed by 1 × Bright-Glo Luciferase Assay reagent (Promega) for at least 2 mins. Luciferase activity was detected with a SpectraMax iD3 Multi-well Luminometer (Molecular devices) within 5 mins. The 50% neutralization dilution titer (NT50) was calculated by GraphPad Prism 8 software with nonlinear regression curve fitting (normalized response, variable slope).

### Spike mediated membrane fusion assay

Vero-E6 or BHK21-hACE2 cells were transfected with plasmids expressing different SARS-CoV-2 spike proteins. Four hours (BHK21-hACE2) or 24 h later (Vero-E6), cells were infected with 100 μl VSV-ΔG-GFP (1 × 10^6^ TCID50/ml) for 4 h. Next, cells were washed once with PBS and supplemented with fresh culture medium. At 8 h post-infection, the nuclei were stained with Hoechst 33342 for 10 mins. Images were captured with a fluorescence microscope (MI52-N).

## Statistical analysis

All data are presented as MEAN ± SD. All statistical analyses were conducted using GraphPad Prism 8. Differences between independent samples were evaluated by unpaired two-tailed t-tests; Differences between two related samples were evaluated by paired two-tailed t-tests. *P* < 0.05 was considered significant. *p < 0.05, **p < 0.01, ***p < 0.001, and ****p < 0.0001.

## Results

**Figure 1.**
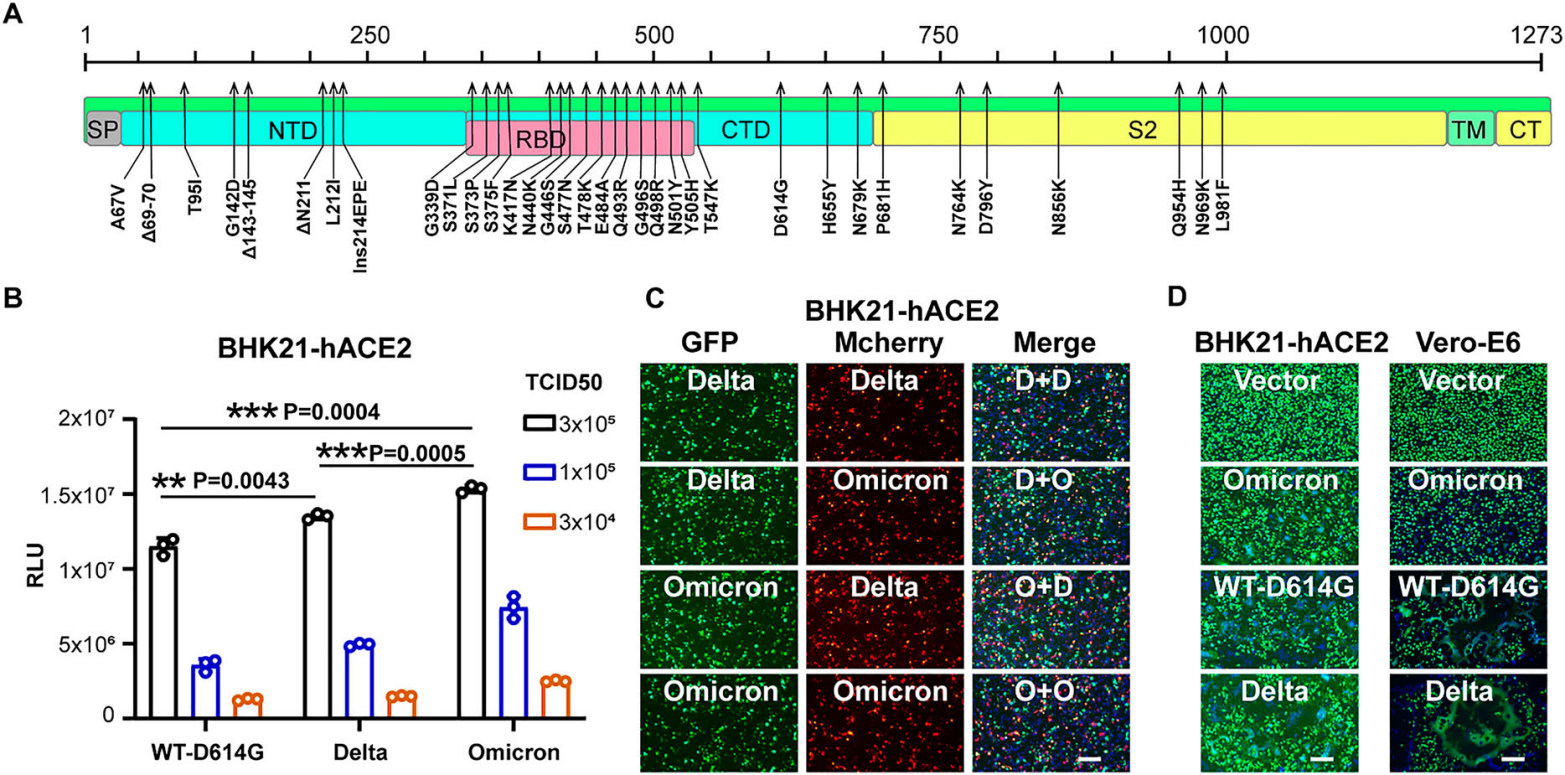
Comparison of the infection features between the Delta and Omicron variants. A. Schematic illustration of the spike mutations of the Omicron variants. Mutations on the Omicron spike protein are indicated by black arrows. SP, signal peptide; NTD, N-terminal domain; RBD, receptor-binding domain; CTD, C-terminal domain; TM, transmembrane domain; CT, cytoplasmic tail. B. Entry efficiency of the SARS-CoV-2 variants in BHK21-hACE2 cells. SARS-CoV-2 pseudoviruses carrying the indicated spike proteins were incubated with the BHK21-hACE2 cells at the indicated TCID50. Luciferase activity was determined at 16 h post-infection (hpi). RLU: Relative Luciferase Unit. Data were presented as Mean ± SD. Statistical significance was determined using unpaired two-tailed t-test, **indicates *P* < 0.01, ***indicates *P* < 0.001. C. Entry competition assay of the Delta and Omicron variants in BHK21-hACE2. The indicated Delta and Omicron pseudoviruses carrying either EGFP or Mcherry reporter gene were mixed with the TCID50 = 1:1 and then incubated with the BHK21-hACE2 for co-infection. At 24 hpi, cell nuclei were stained by Hoechst 33342 for 30 mins, and the images were captured by the microscope. Delta and Omicron were indicated as “D” and “O” in the merged channel, respectively. Scale bars, 200 μm. D. Fusion efficiency of different variants’ spikes in Vero-E6 cells. BHK21-hACE2 and Vero-E6 Cells were transfected with plasmid vector or plasmids expressing spike proteins of the WT-D614G, Delta, or Omicron variants. Next, BHK21-hACE2 cells were infected with VSV-ΔG-GFP at 4 h after transfection, Vero-E6 cells were infected 24 h after transfection. At 8 hpi, cells with or without syncytia formations were treated with Hoechst 33342 for nuclei staining (blue). Images of the green channel and the bright field were captured by the microscope. Scale bars, 200 μm.

We produced SARS-CoV-2 variants pseudoviruses through a well-established glycoprotein deficient Vesicular stomatitis virus (VSV-ΔG) pseudotyping system [17]. The mutations in the Omicron variants are demonstrated in Figure 1A. The Delta variant carries only L452R and T478K mutations in its RBD region, while the Omicron carries as much as fifteen RBD mutations of G339D, S371L, S373P, S375F, K417N, N440K, G446S, S477N, T478K, E484A, Q493R, G496S, Q498R, N501Y, Y505H.

We first compared the infection features between the Omicron and the Delta variants. Pseudovirus (PV) of WT-D614G was used as a reference virus as the D614G is critical for higher viral infectivity, which is also present in the two tested VOC [18]. We conducted the entry assay in the BHK21-hACE2 to characterize the infection mediated by the Omicron variant. The result showed that the infectivity of the Omicron variant is slightly higher than WT-D614G and the Delta variant at the same Median Tissue Culture Infectious Dose (TCID50) (Figure 1B). Viral entry competition assay was conducted using the Delta or the Omicron spike proteins packaged pseudoviruses carrying either green or red fluorescence reporter proteins. The similar green/red ratio and distribution of yellow signal using different spike combinations indicates that the Omicron variant can use human ACE2 at a similar efficiency compared with the Delta variant (Figure 1C).

Interestingly, we observed a big difference in cell–cell fusion efficiency of different variants in both BHK21-hACE2 and Vero-E6 cells. Consistent with the previous report, the Delta variant is more fusogenic than the WT and other VOC [19,20]. In contrast, much fewer cell fusion was observed in the cells expressing the Omicron spike than the WT-D614G spike, indicating that the Omicron variant has reduced fusogenicity (Figure 1D).

**Figure 2.**
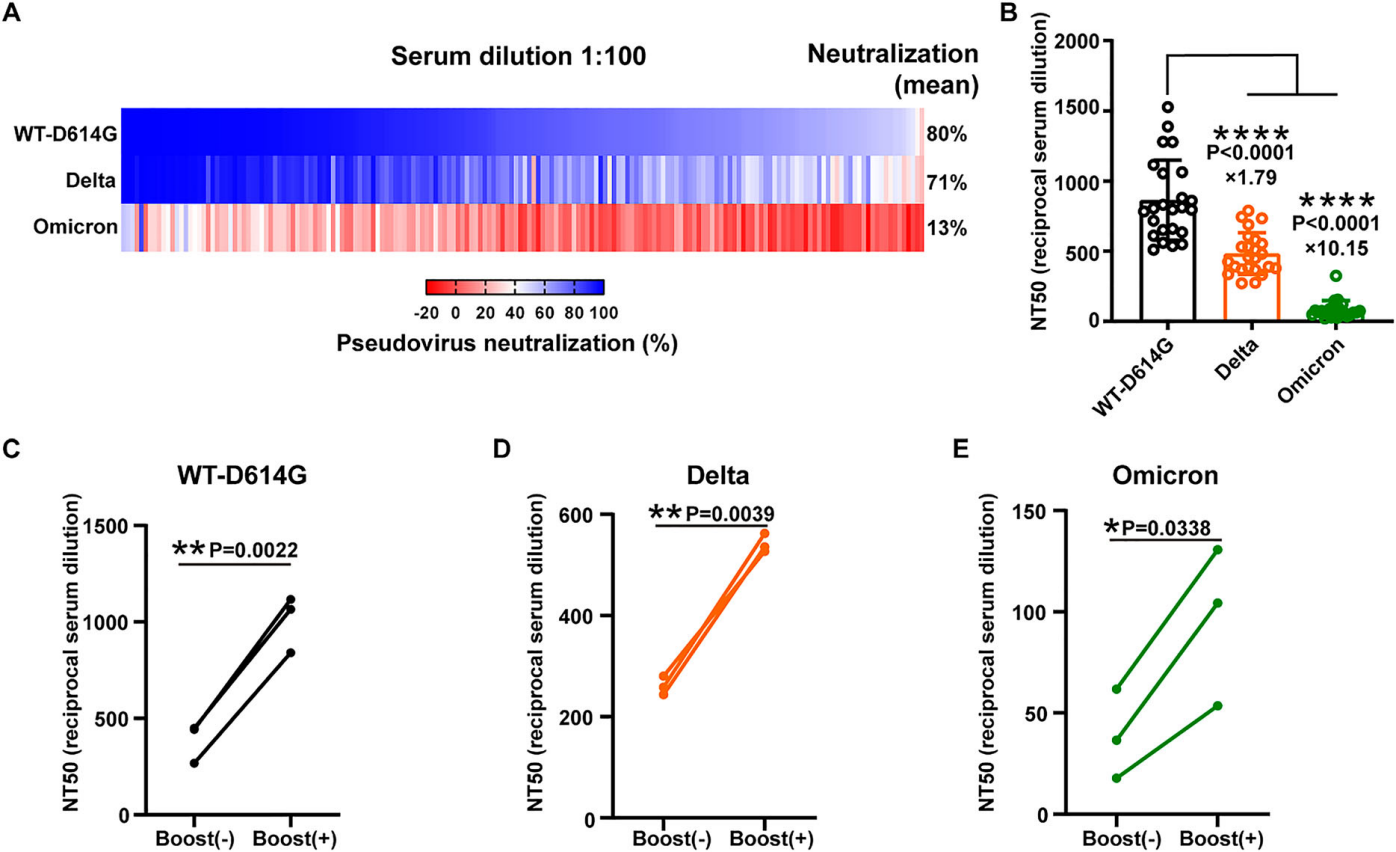
The neutralization efficiency of Wuhan convalescents’ sera against the indicted SARS-CoV-2 variants. A. The heatmap of sera neutralization efficiency against the WT-D614G, Delta, and Omicron variants. Sera from 180 convalescents were 100-fold diluted in the neutralization assay. FBS was used as a negative control. The average sera neutralization efficiency against the WT-D614G, Delta, Omicron were 80%, 71%, 13%, respectively. B. The half neutralization titer of 24 sera against the WT-D614G, Delta, and Omicron variants, respectively. The fold-change of the average NT50 titer was indicated on the data points. Data were presented as Mean ± SD. Statistical significance was determined using a paired two-tailed t-test. ****indicates *P* < 0.0001. C-E. The effect of vaccination on the sera neutralizing activity against the variants in convalescents. The NT50 titer of the sera collected before or after the vaccine boosting was determined against the WT-D614G(C), the Delta (D), and the Omicron (E).

To test the immune escape ability of the Omicron variant, we conducted the pseudovirus neutralization assay in BHK21-hACE2 cells using 180 sera collected from Wuhan convalescents one-year post-infection, all with confirmed anti-SARS-CoV-2 (WT-D614G) activity in our previous study [16]. When the sera were applied at a 100-fold dilution, we observed a dramatic reduction of sera neutralizing activity against the Omicron variant, with the average neutralization of only 13% compared with 80% of WT-D614G. In comparison, the average neutralization of the Delta variant was maintained at 71%. Indeed, only four sera achieved more than half neutralization against the Omicron variant at 100-fold dilution, with the most potent serum showing a 83% inhibition (Figure 2A). To precisely evaluate the reduction of neutralization against the variants, we further tested the half neutralization titer (NT50) of the 24 sera showing the highest activity against the WT-D614G infection. The result showed that, compared with WT-D614G, there is a 10.14 fold decrease of neutralization activity against the Omicron variant, while the decrease against the Delta variant is 1.79 fold (Figure 2B). Notably, sera collected from the three convalescences after three shots of inactivated COVID-19 vaccine showed 2–3 folds boost of neutralization to all the tested viruses. The fold increase of NT50 of Omicron variant is similar to the other two viruses (Figure 2C–E).

## Discussion

Despite the unprecedented speed of COVID-19 vaccine development, the vaccination-based herd immunity strategy is challenged by the recent emergence of the heavily mutated Omicron variant. It has been only one month since the first report of the Omicron, many important questions related to this “new” variant remain to wait for answers. For example, why is it highly transmissible? Why does it seems less pathogenic? How potent can it evade the current anti-SARS-CoV-2 immunity? Our study attempted to address those questions through a well-recognized VSV pseudovirus-based infection system.

For the first question, our data suggest that the Omicron variant is more infectious in BHK21-hACE2 cells, partially explaining its high transmissibility. However, our data do not show a significantly higher efficiency of the Omicron to use human ACE2, which is consistent with a recent study showing an increased affinity of the Omicron RBD binds to ACE2 compared with the original strain [13]. Of note, the entry efficiency could be cell type-specific. Entry assay in other cells types can be performed in future to answer this question. Moreover, the infection system in this study mainly focuses on the single-round viral entry. It is also possible that the Omicron variant has some replication advantages *in vivo*, which can be tested by authentic viruses in an animal model.

The second question is why the omicron infection seems relatively mild or asymptomatic [4]. Although the exact severity of Omicron infection remains to be determined, the milder symptom is consistent with our observation that the Omicron is much less fusogenic, even compared with the original strain. In contrast, the Delta variant can induce a strong cell to cell fusion. This difference may result from the mutation at site P681. P681R has been demonstrated increased fusion in the Delta variant and is associated with the higher pathogenicity of the Delta variant [19,20]. However, the P681H in Omicron might be unfavourable to induce fusion.

For the third question, our sera neutralization results demonstrated the striking immune escape potential of the Omicron variant, which put us on alert to get prepared against the challenge of the Omicron. However, although all sera samples showed dramatically reduced neutralization against the Omicron variant, the neutralizing activity has not been completely wiped out. The retained activity might be helpful to reduce the infection chance or disease severity. The convalescents should also be vaccinated, as our results indicate the sera neutralizing activity against the Omicron variant significantly increased after the three shots of inactivated-vaccine. An Omicron-specific booster shot could be more helpful as a recent study indicate the omicron variant is less sensitive to the booster shot [21].

So far, the Delta variant remains the global dominant strain, but the Omicron variant may take its place soon, considering its recent surge in global infection cases. The higher transmission efficiency, more asymptomatic patient ratio, and strong immune evasive ability may contribute to the rapid spreading of the Omicron variant. Notably, whether the Omicron variant could become more pathogenic through mutating or recombination with other strains remains open. The situation is becoming evident that we are facing a long-lasting battle against the sophisticated SARS-CoV-2 virus.

In summary, our study gives further evidence that the Omicron variant represents a “brand-new” SARS-CoV-2 variant with different infection features and is much more immune evasive than the Delta variant. The effectiveness of anti-SARS-CoV-2 immunity in most people developed by prior infection or vaccination should be re-evaluated facing the Omicron variant. The convalescents should also be vaccinated, and the vaccine recipient should get booster shots to enhance their immunity against the emerging variants such as the Omicron. Active measures should be taken to update the current vaccines and develop the Omicron-specific/broadly neutralizing antibodies or other cross-variant antiviral therapeutics.

## Data Availability

All data are available in the manuscript.
